# Multi-institutional validation of AI models for classifying urothelial neoplasms in digital pathology

**DOI:** 10.1038/s41598-025-21096-1

**Published:** 2025-10-24

**Authors:** Jun Young Park, Jisup Kim, Young Jae Kim, Sung Hyun Kim, Chi Sung An, Kwang Gi Kim, Chan Kwon Jung

**Affiliations:** 1 Deptartment of Translational-Clinical Medicine, Gachon University, Incheon, Republic of Korea; 2 Department of Pathology, Gil Medical Center, Gachon University College of Medicine, Incheon, Republic of Korea; 3 Gachon Biomedical & Convergence Institute, Gachon University Gil Medical Center, Incheon, Republic of Korea; 4 Department of AI Data, National Information Society Agency (NIA), Deagu, Republic of Korea; 5 Urban Datalab, Electronics and Telecommunications Research Institute Convergence Center, Deajeon, Republic of Korea; 6 Medical Devices R&D Center, Gachon University Gil Medical Center, Incheon, Republic of Korea; 7 Department of Hospital Pathology, Seoul St. Mary’s Hospital, College of Medicine, The Catholic University of Korea, Seoul, Republic of Korea

**Keywords:** Cancer, Computational biology and bioinformatics, Oncology, Urology

## Abstract

This study proposes a deep learning approach for classifying normal, noninvasive, and invasive urothelial neoplasms via digitized histopathologicalimages. Despite many artificial intelligence (AI) models for cancer diagnosis, few focus on bladder lesions or differentiate between these critical categories. We developed convolutional neural networks (CNNs) and transformer-based models, which were trained on 12,500 whole-slide images (WSIs) from five institutions, with preprocessing steps including stain normalization and patch extraction. Fivefold cross-validation was used for evaluation against expert-annotated labels. Among tested models, EfficientNet-B6 achieved the highest performance, with an accuracy of 0.913 (95% confidence interval (CI), 0.907–0.920), sensitivity of 0.909 (95% CI, 0.904–0.914), specificity of 0.956 (95% CI, 0.953–0.960), F1-score of 0.906 (95% CI, 0.901–0.911), and an area under the receiver operating characteristic curve (AUC) of 0.983 (95% CI, 0.982–0.984). These results demonstrate the effectiveness and generalizability of AI-based bladder cancer classification.

## Introduction

Bladder cancer is a common malignancy of the urinary tract, and is the tenth most frequently diagnosed cancer worldwide^1,2^. According to the 2020 GLOBOCAN database, 573,000 new cases and 213,000 deaths occurred worldwide, corresponding to age-standardized ratees of 5.6 and 1.9 per 100,000, respectively^3^. The global burden of bladder cancer is projected to rise substantially, with expected new cases reaching 991,000 new cases (a 72.8% increase) and 397,000 deaths (an 86.6% increase) by 2040, with the most largest increases in Western Pacific region.

Initial diagnostic evaluation typically includes cystoscopy, urinary tract imaging, and urine analysis^4^. The gold standard for histopathological diagnosis relies on microscopic examination of hematoxylin and eosin (H&E)-stained tissue sections by pathologists^5^. In routine clinical practice, these tissue samples are obtained through transurethral resection of bladder tumor (TURBT) or radical cystectomy, with subsequent processing, staining, and digitization into whole-slide images (WSIs) for detailed review^5^. Digital pathology, which converts glass slides into high-resolution digital images, is increasingly used to facilitate remote consultation, archiving, quantitative analysis, and integration into computer-aided diagnostic systems^6^. Despite being the cornerstone of diagnosis, microscopic examination of pathology slides is labor-intensive, time-consuming, and subject to interobserver variation^7^. Repeated evaluations under high workloads can lead to diagnostic fatigue and errors, particularly when distinguishing morphologically overlapping entities, such as normal urothelium, noninvasive urothelial neoplasms, and invasive urothelial carcinoma^8^. To address these challenges, artificial intelligence (AI) and deep-learning approaches have been increasingly investigated for their potential to increasediagnostic accuracy and efficiency in digital pathology^9^.

Previous studies demonstrated the utility of AI models for cancer classification viaWSIs. For example, an ensemble deep-learning model for breast cancer classification demonstrated exceptionally high performance metrics, including an accuracy of 99.5%, precision of 0.99, recall of 0.99, and an F1 score of 0.99 on the BreakHis dataset^10^. Similarly, convolutional neural networks (CNN)-based models applied to gastric cancer have yielded high classification accuracy, reaching up to 98.5%, along with meaningful segmentation performance^11^. In addition cancer detection, a previous study classified tumors into finer categories, such as renal cell carcinoma subtypes (e.g., clear cell, papillary, and chromophobe), as well as broader categories of renal tissue, including non-neoplastic tissue, benign tumors, and malignant tumors^12^. The study achieved high classification accuracy through generative adversarial networks (GAN)-based stain normalization and optimized architectures incorporating multiple-instance learning (MIL).

In the context of bladder cancer, previous studies have primarily focused on distinguishing noninvasive from invasive tumors (e.g. pTa vs. pT1) or grading (e.g., low-grade vs. high-grade) in biopsy or TURBT specimen on single institutional basis. For instance, machine learning models using handcrafted features extracted via ImageJ and CellProfiler achieved an accuracy of 91–96% in distinguishing noninvasive (pTa) and invasive (pT1) tumor, while CNN models reached approximately 84% accuracy^13^. Another study employing MIL for grading non-muscle invasive bladder tumor (pTa/pT1) achieved an F1 score of 0.85 based on 300 WSIs^14^. However, utilizing biopsy or TURBT have inherent limitations, as they are often fragmentated or tangentially orientated and do not capture the full anatomic layer of the urinary bladder, especially deep layers (muscularis propria and perivesical soft tissue) as well as adjacent normal tissue^15^. In this study, we sought to construct a comprehensive histopathological model of the urinary bladder that extends beyond the tumor-focused and mucosa/submucosa-restricted nature of biopsy or TURBT specimens, utilizing multi-institutional data to improve generalizability and applicability across diverse clinical settings. Our approach encompassed a broad spectrum of histological categories, including normal tissue, noninvasive urothelial neoplasms, and invasive urothelial carcinoma, while capturing the full anatomical layers of the urinary bladder—urothelium, lamina propria, muscularis propria (detrusor muscle), and surrounding stroma. We developed a pathological deep-learning model trained on multi-institutional datasets, including radical cystectomy specimens, to classify urinary bladder tissue on WSIs into three broad categories: normal, noninvasive urothelial neoplasms, and invasive urothelial carcinoma. The model exhibited robustness and generalizability, with the potential to support pathologists in accurate diagnosis and clinical decision-making in real-world practice.

## Results

A total of 12,500 WSIs were included in this study, including 1,500 normal bladder tissues, 5,500 noninvasive urothelial neoplasms, and 5,500 invasive urothelial neoplasms. WSIs classified as invasive urothelial carcinomas were derived from patients with tumors invading to the lamina propria (pT1:2,575 [46.82%]), muscularis propria (pT2:1,751 [31.84%]), perivesical soft tissue (pT3:799 [14.53%]), and extravesical tissues (pT4:375 [6.81%])^16^. Most of the WSIs classified as noninvasive urothelial neoplasms were derived from patients with noninvasive papillary urothelial carcinoma (pTa, 4,728 cases; 85.96%) and urothelial carcinoma in situ (pTis, 712 cases; 12.95%). A small proportion within this class originated from noninvasive components of invasive urothelial carcinomas (58 from pT1 [1.05%], 1 from pT2 [0.02%], 1 from pT3 [0.02%], and 0 from pT4).

**Fig. 1:**
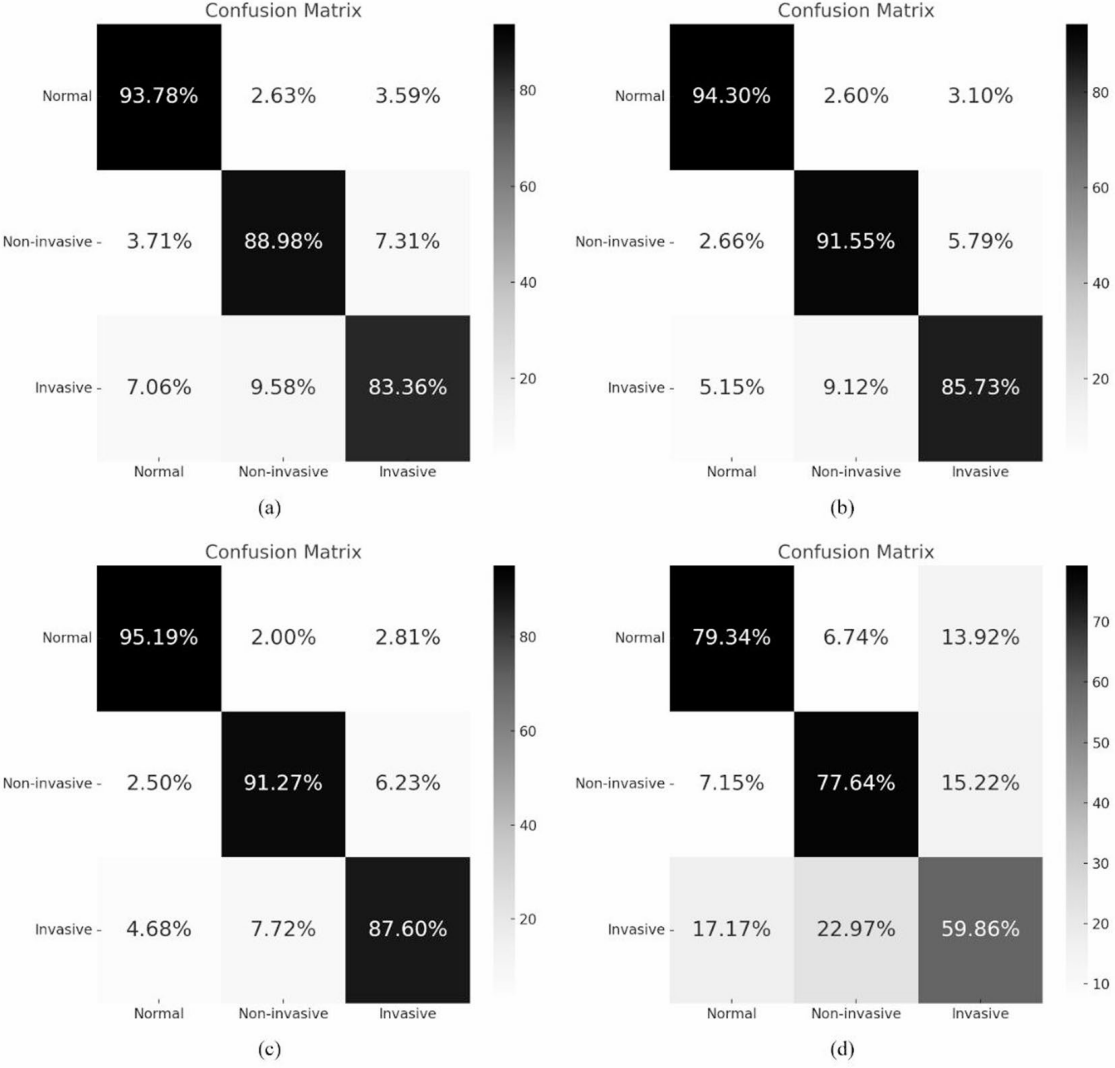
5-Fold confusion matrix results for four deep learning classification models (row-wise normalized; diagonal values indicate per-class sensitivity): (**a**) ResNet-50, (**b**) DenseNet-121, (**c**) EfficientNet-B6, (**d**) ViT.

In this study, the classification performance of four deep-learning models was evaluated using 5-fold cross-validation to assess their ability to distinguish between normal, noninvasive, and invasive classes. Figure 1 presents the aggregated confusion matrix from 5-fold cross-validation. The confusion matrices obtained from each fold are summed to yield a single overall matrix. Among the evaluated models, EfficientNet-B6 demonstrated superior performance overall. Although per-class sensitivity for the noninvasive category was marginally lower than DenseNet-121 by 0.28%, EfficientNet-B6 exceeded DenseNet-121 by 1–2% in the other two classes and achieved the best overall performance among all models. The confusion matrices obtained from each fold were aggregated to yield a single overall matrix. Among the evaluated models, EfficientNet-B6 (c) demonstrated the best performance. Although its per-class sensitivity for noninvasive category was marginally lower than DenseNet-121 (b) by 0.28%, it surpassed DenseNet-121 by 1–2% in the other two classes and consistently outperformed ResNet-50 (a) and ViT (d) across all categories.

**Fig. 2:**
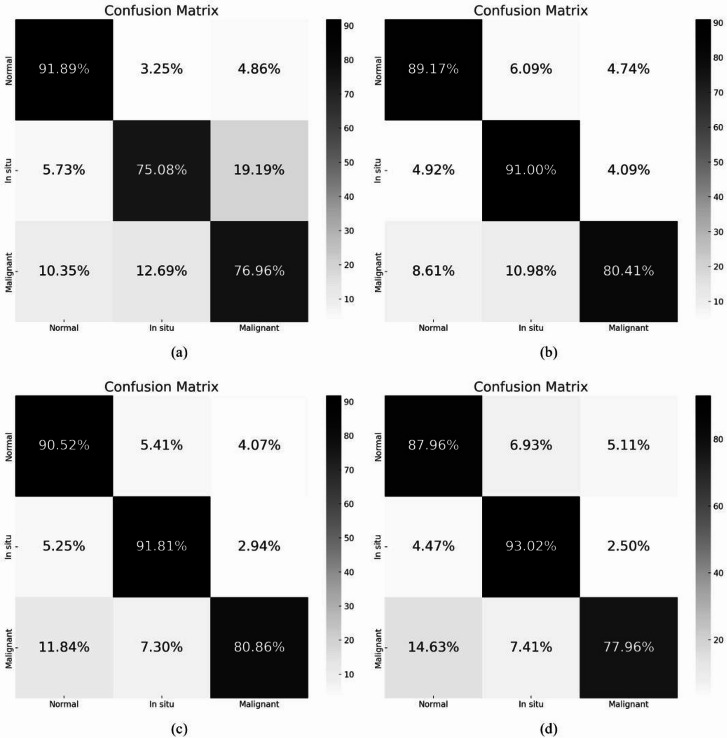
Classification performance by tumor invasion depth within the malignant class: (**a**) pT1 subgroup, (**b**) pT2 subgroup, (**c**) pT3 subgroup, (**d**) pT4 subgroup.

Figure 2 presents the classification performance results for distinguishing normal and non-invasive classes after stratifying the malignant cases according to their invasion depth (pT1–pT4). Specifically, Fig. 2a, b and c, and 2d correspond to the pT1, pT2, pT3, and pT4 subgroups, respectively. These results demonstrate that the models were able to achieve adequate classification performance even when the malignant class was subdivided by invasion depth, suggesting their potential applicability to tumors with varying degrees of invasion.

**Fig. 3:**
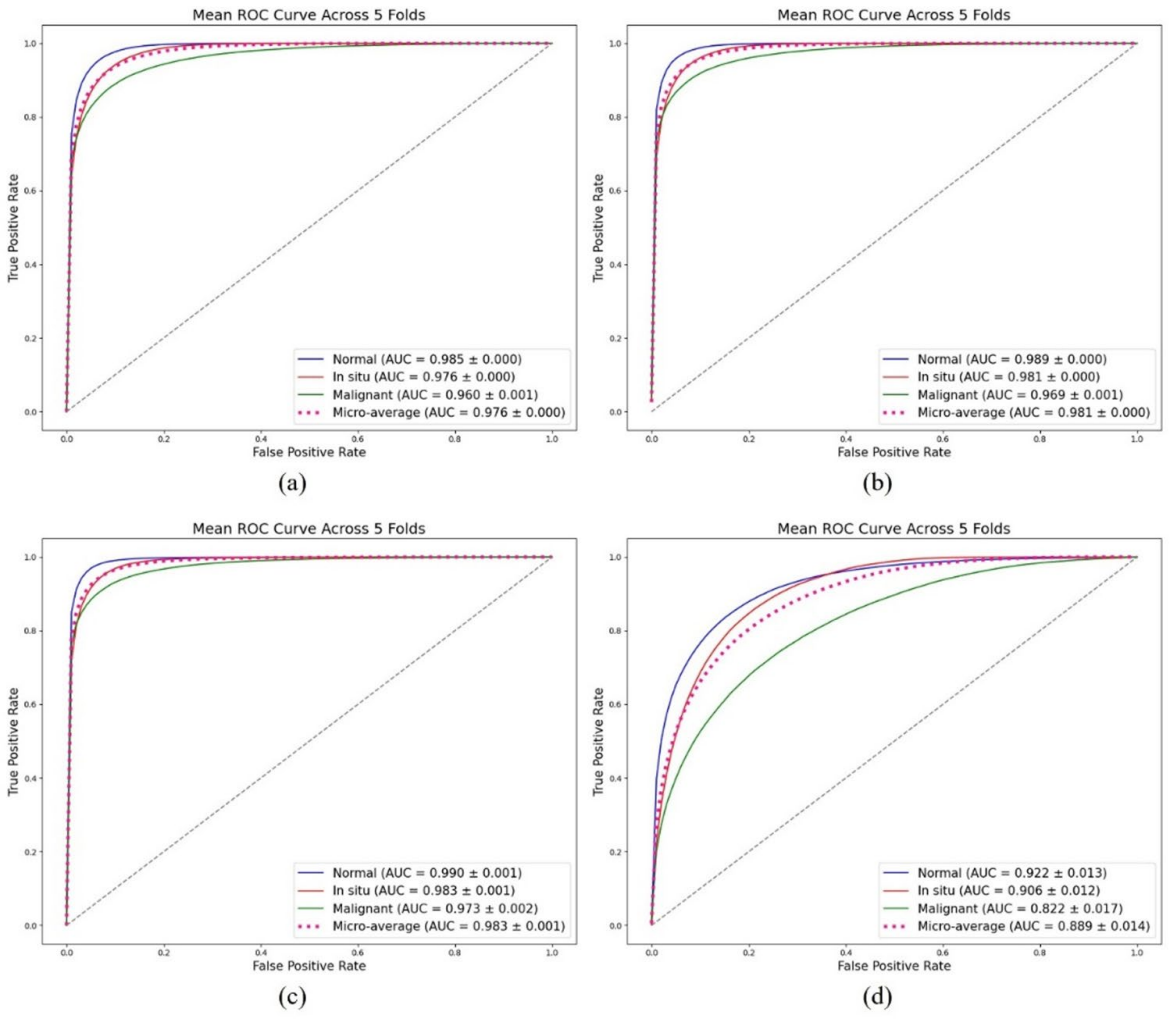
5-Fold ROC curve results for four deep learning classification models: (**a**) ResNet-50, (**b**) DenseNet-121, (**c**) EfficientNet-B6, (**d**) ViT.

Table 1 presents the average test set performance metrics with 95% confidence intervals (CIs) across five-folds. Among the four models (ResNet-50, DenseNet-121, EfficientNet-B6, and ViT), EfficientNet-B6 consistently achieved the highest an accuracy of 0.913 (95% CI, 0.907–0.920), a sensitivity of 0.909 (95% CI, 0.904–0.914), a specificity 0.956 (95% CI, 0.953–0.960), and an F1-score 0.906 of (95% CI, 0.901–0.911), followed by DenseNet-121 and ResNet-50, while ViT demonstrated markedly lower performance across all metrics. These findings suggest that CNN-based architectures, particularly EfficientNet-B6, provide superior performance compared with the transformer-based model in the histologic classification of urinary bladder pathology. Figure 3 shows the average receiver operating characteristic (ROC) curves from 5-fold cross-validation of the four deep-learning classification models. As shown in the figure, EfficientNet-B6 had the highest AUC values across all classes compared with the other models: normal (AUC 0.990), noninvasive (corresponding to in situ in the figure, AUC 0.983), and invasive (corresponding to malignant in the figure, AUC 0.973). The highest micro-average AUC of 0.983 was recorded. By contrast, the ViT model had the lowest AUC values for each class as well as the lowest micro-average AUC.

**Table 1:** ResNet-50, DenseNet-121, EfficientNet-B6, and ViT 5-fold cross validation results for classification.

Model	Accuracy (95% CI)	Sensitivity (95% CI)	Specificity (95% CI)	F1-score (95% CI)
ResNet-50	0.887 (0.884–0.889)	0.885 (0.882–0.888)	0.943 (0.942–0.944)	0.886 (0.884–0.888)
DenseNet-121	0.905 (0.903–0.907)	0.902 (0.898–0.906)	0.952 (0.951–0.953)	0.901 (0.896–0.906)
EfficientNet-B6	0.913 (0.907-920)	0.909 (0.904–0.914)	0.956 (0.953–0.960)	0.906 (0.901–0.911)
ViT	0.722 (0.696–0.749)	0.718 (0.706–0.730)	0.861 (0.848–0.874)	0.720 (0.695–0.745)

**Fig. 4:**
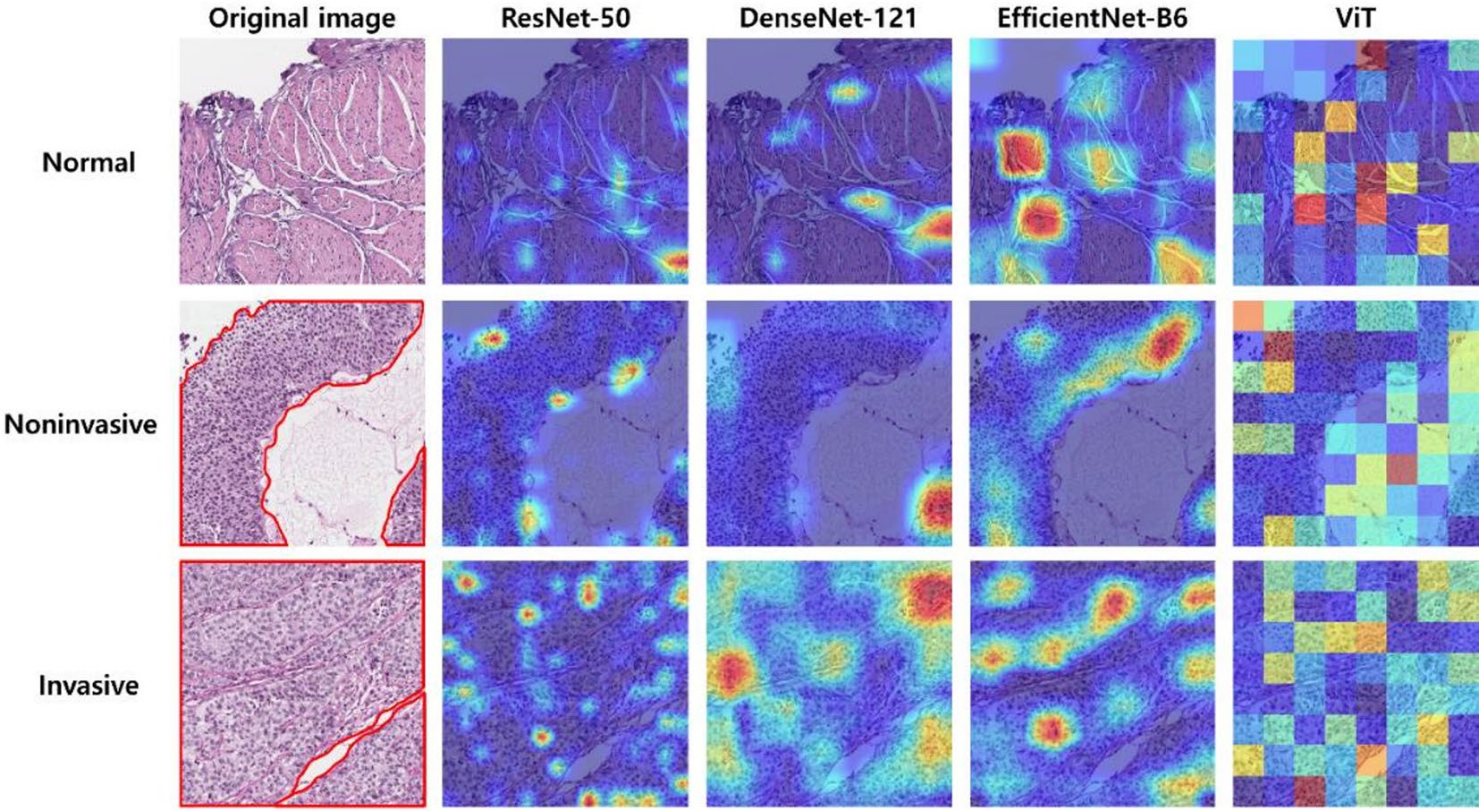
CAM visualization results of ResNet-50, DenseNet-121, EfficientNet-B6, and ViT models.

Figure 4 shows the CAM for each model in the form of heatmaps across the three classes: normal, noninvasive, and invasive. As shown in the figure, the EfficientNet-B6 and DenseNet-121 models consistently highlighted broader, pathologically relevant regions that aligned with diagnostic features. ResNet-50 also focused on appropriate areas but tended to concentrate on very small, localized points, potentially overlooking the broader architectural feature required for optimal classification. In contrast, ViT produced scattered activations, frequently highlighting regions corresponding to unrelated classes or even empty background areas, underscoring its limited pathological relevance. For noninvasive cases, the three CNN-based models focused on the tumor boundary, an area consistently emphasized by pathologists in diagnostic practice, whereas in invasive cases activations generally spread across broader tumor regions without distinct focal concentration. Overall, DenseNet-121 and EfficientNet-B6 provided the most reliable and interpretable CAM patterns, followed by ResNet-50, whereas ViT performed the poorest.

**Fig. 5:**
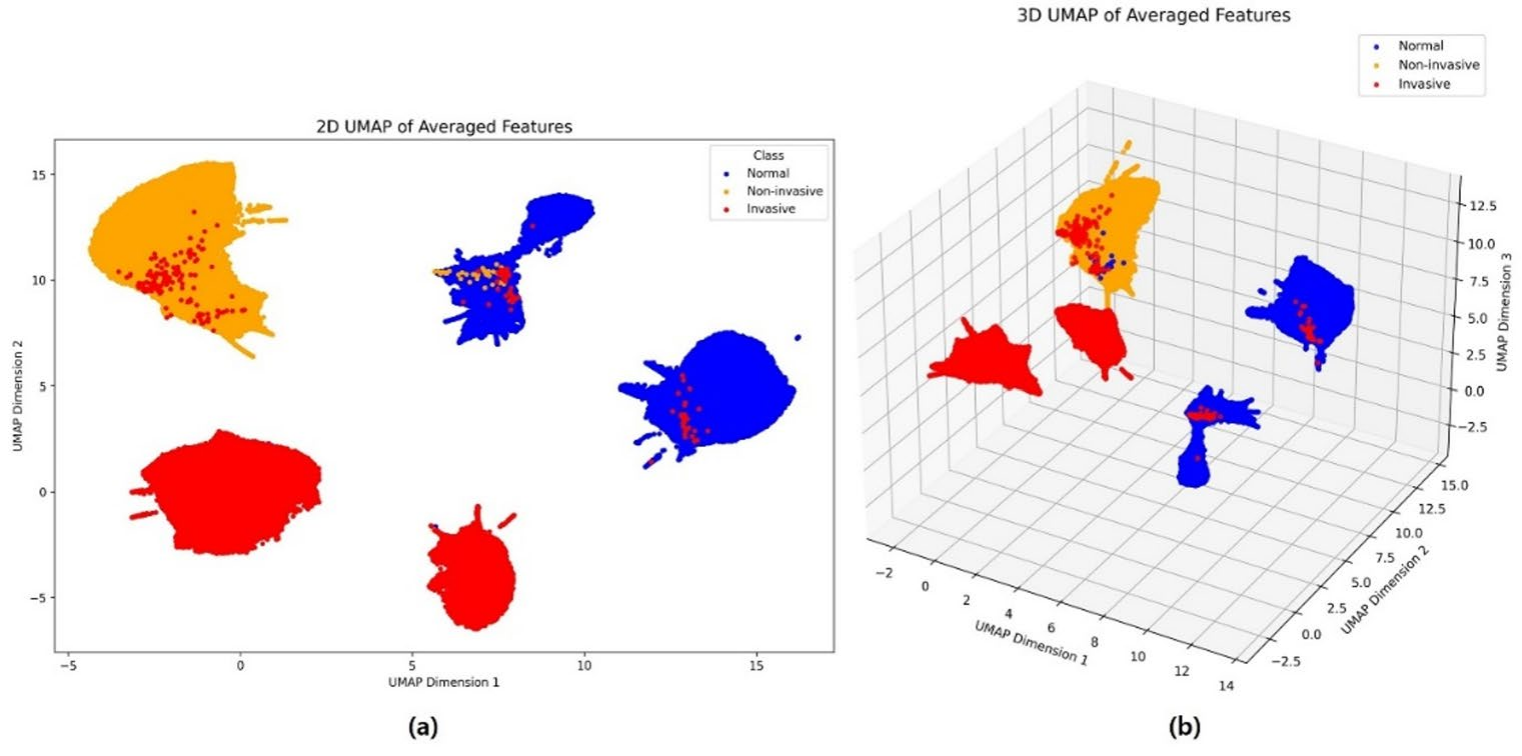
UMAP-based visualization of feature representations from the EfficientNet-B6 model, illustrating the distributions of normal, noninvasive, and invasive classes: (**a**) 2D UMAP visualization of the normalized feature representations; (**b**) 3D UMAP visualization of the normalized feature representations.

Figure 5 presents the Uniform manifold approximation and projection (UMAP) visualization of the normalized features extracted from the EfficientNet-B6 model for the normal, noninvasive, and invasive classes. Figure 4a shows the two-dimensional UMAP projection based on normalized features, whereas Fig. 4b shows the corresponding three-dimensional projection. This visualization was performed as the final step of model validation via the EfficientNet-B6 model, which demonstrated relatively high performance. As shown in the figure, the feature distributions for each class were well separated, indicating that the model effectively captured class-specific representations.

## Discussion

In this study, four classification models, ResNet-50, DenseNet-121, EfficientNet-B6, and ViT, were used to classify normal, noninvasive, and invasive categories. The labeled regions corresponding to the normal, noninvasive, and invasive classes were defined by board-certified pathologists on the basis of the WSIs data. Patches were extracted from these annotated regions and used to train and validate the four deep-learning classification models. Model training and validation were performed via 5-fold cross-validation to assess classification performance. The evaluation metrics included the accuracy, precision, F1 score, and AUC. The experimental results showed that the CNN-based models achieved a performance above 0.85, whereas the transformer-based models demonstrated a comparatively lower performance. Among them, EfficientNet-B6 achieved the highest performance, with an accuracy of 0.913, sensitivity of 0.909, specificity of 0.956, F1-score of 0.906, and AUC of 0.983.

The EfficientNet-B6 model achieved the highest predictive performance among all the models tested, demonstrating superior results across all evaluation metrics. EfficientNet-B6 dynamically adjusted the depth and width of the model according to the input image size via a compound scaling method that simultaneously considers depth, width, and resolution. In this study, the input size of the patch data was 512 × 512. Among the EfficientNet series ranging from B0 to B7, the B6 model with a recommended input size of 528 × 528 was the most closely aligned with the experimental data. Therefore, this model was selected for use in the present study. Compared with the other models, EfficientNet-B6 provided optimal depth and width tailored to the patch data, which contributed to its superior performance. Model depth and width are critical factors in network design. As the number of layers (depth) in a model increases, simply adding more layers does not necessarily lead to improved performance; instead, it is important to balance network design by jointly scaling depth, width, and resolution, as exemplified by the EfficientNet architecture^17^. Thus, EfficientNet-B6 appears to be a well-designed model for this task. The findings of this experiment provide a strong foundation for future work aimed at designing optimized models for a more effective classification of normal tissue and invasion status via EfficientNet-B6. Moreover, this study is significant in that it enhances the potential clinical application of the model.

However, the ViT model demonstrated the lowest performance among all the tested models. Although the dataset used in this study comprised more than 100,000 images in terms of absolute quantity the unique characteristics of medical images may have limited the ability of the transformer architecture to leverage its strengths. Therefore, the suboptimal performance of ViT observed in this study is likely attributable not to the limitation of dataset size alone, but rather to the inherent nature of pathological imaging data arising from the clonal nature of neoplasia^18^ and the challenges involved in optimizing class-level representations. These results highlight the importance of aligning data characteristics with an appropriate deep-learning model, particularly for pathological image classification. The ViT model divides the input image into patches and predicts the final class by aggregating the features extracted from these patches. The relatively poor performance of the ViT model indicates a limitation in its ability to capture cytological features because it tends to focus on global features across patches. An examination of the confusion matrices across folds for the ViT model revealed a tendency toward polarization between the normal and invasive classes, unlike the other models. This suggests that when images are divided into very small patches, it becomes difficult to assess invasiveness as an inherent histological concept. As observed in the extracted patches, determining invasiveness requires an understanding of tissue-level infiltration, which is difficult to assess from a purely cytological viewpoint. By contrast, CNN-based models are likely to perform better because they process patch data without further dividing the image, allowing them to better capture the relevant spatial and morphological features necessary for accurate classification.

In the visual evaluation conducted in this study, the EfficientNet-B6 model demonstrated a clear advantage over the other models in terms of utilizing anatomically accurate regions as visual evidence, as confirmed by the CAM results. In the normal class, the model focused on relatively broad and accurate tissue regions, and in the noninvasive class, it successfully identified relevant pathological areas. By contrast, the ViT model relied on patchwise attention scores, which highlighted less precise regions, likely contributing to its relatively lower classification performance. For the final visual validation, UMAP was employed with normalized features extracted from the last layer of the high-performance EfficientNet-B6 model. As illustrated in the UMAP visualization, the features of the three classes were generally well-clustered. However, some invasive data points appeared within the noninvasive cluster, and both invasive and noninvasive data were partially embedded in the normal cluster. These overlaps may be attributed to the intrinsic difficulty in distinguishing between the invasive and noninvasive classes, as some borderline cases present ambiguous histological features. Additionally, because the classification was performed on patch-level data extracted from annotated regions, patches located at the edges of invasive areas may have been included in the noninvasive class. Similarly, patches extracted near the boundaries of normal regions may contain features from other classes or may lack sufficient pathological cues. These findings suggest that patch-level classification may suffer from limitations in accurately capturing invasive characteristics owing to partial or mixed features within individual patches. Such limitations can potentially be addressed by employing models trained on entire tissue regions rather than on isolated patches, thereby providing a more holistic understanding of the histological context.

A key focus of this study was to explore the feasibility of classifying invasive and noninvasive cancers. Unlike normal tissues, cancerous regions typically consist of abnormal cells that can be visually identified based on cell density and the presence of tumor masses. However, invasiveness is more effectively observed histologically at low magnification than by cytological examination of individual cells. By leveraging this characteristic, we extracted and utilized 10× magnified patches corresponding to low magnifications for training and validation. However, one limitation of this study is that only 10× magnification patch data were used, which restricts the analysis of invasiveness at other magnification levels. Additionally, because the WSIs data were acquired at 20× magnification, the number of extractable patches was inherently limited. Another limitation of this study is that we were unable to apply recently proposed pretrained foundation models for WSI classification. We believe that the use of such models could have led to improved performance^19^. In particular, we believe that appropriately fine-tuning transformer-based foundation models could partially overcome the limitations of the ViT model observed in this study^20^. Furthermore, due to the imbalance in the number of cases across institutions, we were unable to independently analyze the performance by institution. This remains another limitation of our study.

In future studies, collecting WSIs data at higher magnifications could enable the analysis of invasiveness across multiple magnification levels, helping to identify the magnification that provides the most accurate classification. Recent studies have also examined breast cancer by extracting patches at various magnifications to determine which level yields the highest performance in distinguishing normal from cancerous tissue^21^. By utilizing this type of analytical approach, it is expected that a more detailed analysis of bladder cancer will be possible. In addition, as a potential future research direction, the extraction of features from multiscale patch data to identify cancerous regions may be feasible. Recent studies have explored this by using patches at 5×, 10×, and 20× magnifications as multiple inputs to segment invasive tumor and carcinoma in situ regions^22^. Utilizing such an analytical approach to segment invasive tumor in patients with bladder cancer could help address the limitations identified in this study.

This study used data collected from five university hospitals for model training and validation. In the future, by collecting additional data from other institutions and continuously refining the training and validation processes, an optimized model architecture for bladder cancer can be developed. From a clinical perspective, deep-learning approaches have the potential to reduce the time and cost burdens on pathologists. Furthermore, advanced deep-learning classification models may evolve into systems capable of automatically identifying bladder cancer regions and analyzing their invasiveness.

## Methods

**Fig. 6:**
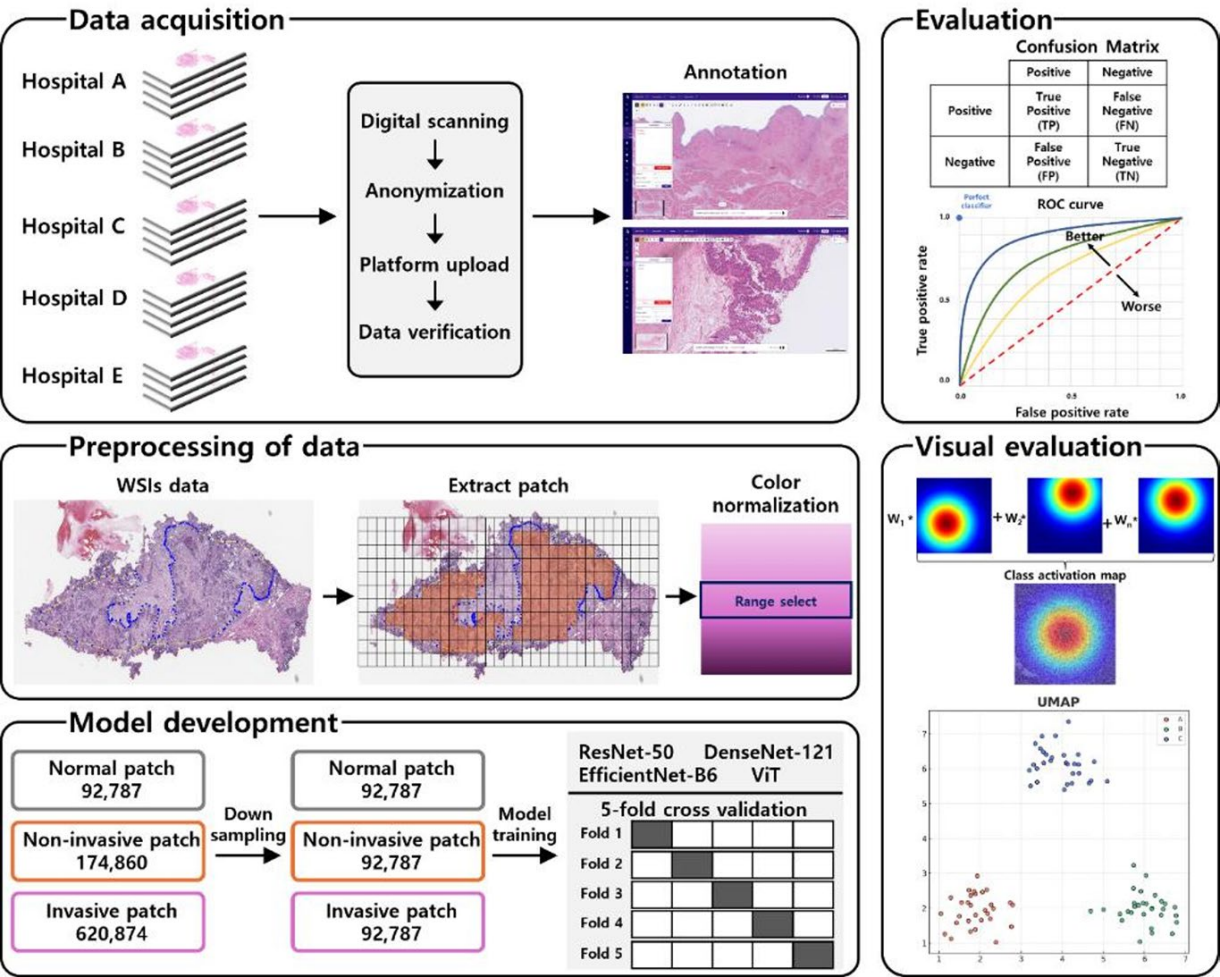
Overall workflow of the proposed method for classifying normal, noninvasive, and invasive cases in bladder cancer pathology data.

Data collected from five university hospitals were used to train a deep-learning model to classify cases into normal, noninvasive, and invasive categories. Figure 6 presents the overall workflow of the study, which consists of five main components: data acquisition, data preprocessing, model development, evaluation, and visual evaluation. This study was performed in accordance with the principles of the Declaration of Helsinki. Informed consent was obtained from all participants and their legal guardians prior to inclusion in the study. The study was approved by the Institutional Review Board (IRB) of Seoul St. Mary’s Hospital (KC23RNDI0458), St. Vincent’s Hospital (VC23SIDI0187), Uijeongbu St. Mary’s Hospital (UC23SNSI0078), Ajou University Hospital (AJOUIRB-DB-2023-405), and Korea University Anam Hospital (2023AN0243).

### Data

Histopathological data were retrospectively collected from five university hospitals over an eight-month period from May to December 2023. The dataset comprised 12,500 H&E-stained WSIs obtained from patients diagnosed with or suspected of having bladder cancer, including those who had undergone radical cystectomy. Among these, 1,500 samples represented normal tissue, 5,500 were classified as noninvasive urothelial neoplasms and 5,500 were calssified as invasive urothelial carcinoma (pT1–4). Noninvasive urothelial neoplasms includ papillary urothelial neoplasms with low malignant potential, noninvasive papillary urothelial carcinoma (pTa), and urothelial carcinoma in situ (pTis).

All the collected pathology slides were digitized at high resolution, enabling visualization at up to 20× magnification. Digital scanning was performed via three whole-slide scanners: Hamamatsu NanoZoomer S360 (Hamamatsu Photonics, Japan), Aperio GT450 (Leica Biosystems, USA), and Pannoramic 250 Flash III (3DHISTECH, Hungary).

This study aimed to classify histological images into three diagnostic categories: normal tissue, noninvasive urothelial neoplasms, and invasive urothelial carcinoma. The “normal” class encompassed all histologically unremarkable bladder tissues, including the urothelium, lamina propria, muscularis propria (detrusor muscle), and surrounding stroma. Pathologically relevant areas corresponding to normal bladder tissue, noninvasive urothelial neoplasms, and invasive urothelial carcinomas were annotated by board-certified pathologists from each of the five participating university hospitals.

**Fig. 7:**
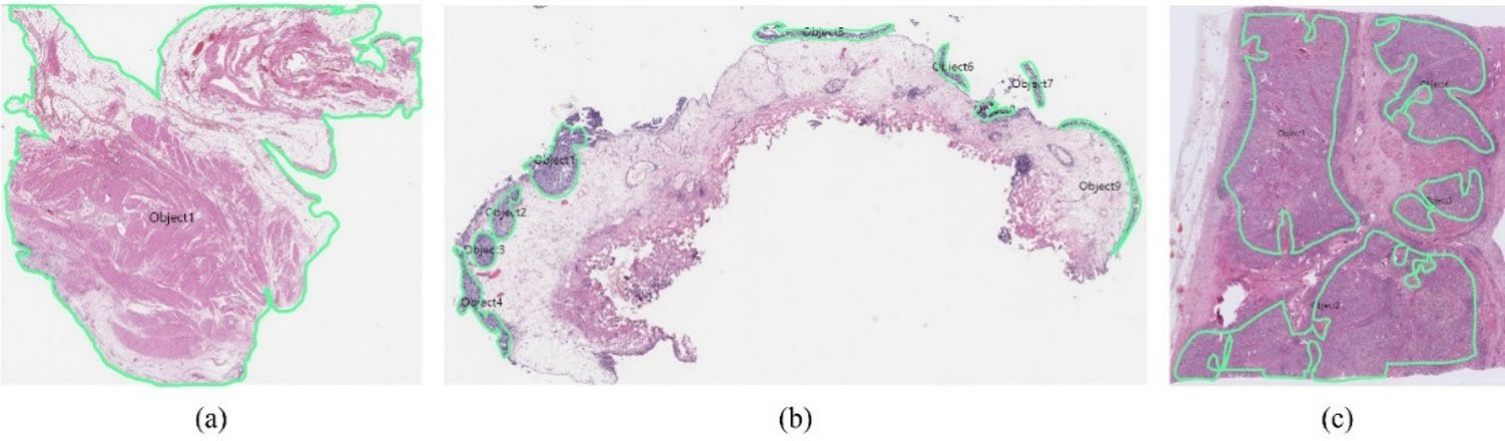
Representative examples of pathologist-annotated regions on whole-slide images: (**a**) normal, (**b**) noninvasive urothelial neoplasm, and (**c**) invasive urothelial carcinoma.

To manage the large file sizes inherent to WSIs data, a cloud-based digital pathology platform, MeDIAuto (UrbanDataLab, Seoul, Korea), was used to streamline image upload, remote access, and labeling. With this platform, pathologists labeled representative areas corresponding to normal, noninvasive urothelial neoplasms, and invasive urothelial carcinomas. A cross-review process was implemented to ensure annotation reliability, whereby the pathologists reviewed and validated each other’s labels. Figure 7 illustrates the annotated regions for each diagnostic class: normal (Fig. 7a), noninvasive urothelial neoplasm (Fig. 7b), and invasive urothelial carcinoma (Fig. 7c).

### Research environment

This study was conducted in a Python-based environment (version 3.9.0) on Ubuntu 20.04 Long Term Support (Canonical, London, United Kingdom (UK)) using PyTorch^23^ (version 2.3.0), Computed Unified Device Architecture (CUDA) (version 11.8), and the CUDA Deep Neural Network Library (version 8.6.0). Deep-learning training was performed using an NVIDIA A100 40 GB graphic processing unit (GPU) (NVIDIA, Santa Clara, CA, USA). MedCalc statistical software (version 14.8.1, MedCalc Software Ltd, Ostend, Belgium) was used to analyze whether there were statistically significant differences between groups^24^. To handle and preprocess the WSIs data, Slideio (version 2.2.0), Staintools (version 2.1.2), OpenCV-Python^25^ (version 4.8.1.78), and Matplotlib (version 3.7.1) were used. Scikit-learn^26^ (version 1.2.2) was used to evaluate the model performance.

### Data preprocessing

WSIs data are significantly larger in both file size and resolution than those of other medical imaging modalities, posing challenges for computational efficiency and model training. Resizing full-resolution WSIs often leads to the loss of critical histological details, particularly in tasks that require accurate interpretation of tissue architecture and structure. To address these limitations, a patch-based extraction strategy was implemented.

Noninvasive urothelial neoplasms and invasive urothelial carcinomas are primarily distinguished by invasion of the lamina propria. Therefore, it is crucial to focus on capturing architectural features, rather than cellular-level atypia. The image patches were extracted at a magnification of 10×, which provided an optimal balance between the resolution and the histological context. Each patch was then resized to 512 × 512 pixels to standardize the input dimensions for training the deep-learning models.

**Fig. 8:**
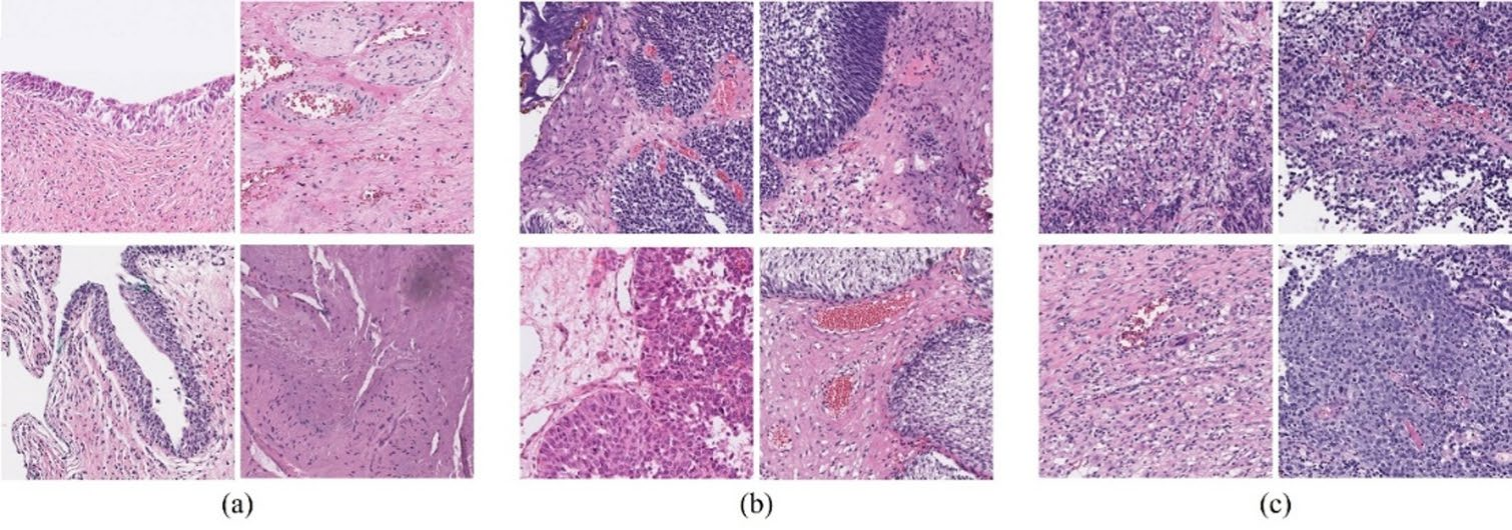
Representative image patches extracted from whole-slide images: (**a**) patches from normal bladder tissue, (**b**) patches from noninvasive urothelial neoplasms, and (**c**) patches from invasive urothelial carcinoma.

A total of 888,341 image patches were generated, including 92,787 patches from normal tissue, 174,680 from noninvasive urothelial neoplasms, and 620,874 from invasive urothelial carcinoma. Representative examples of each diagnostic category are shown in Fig. 8: (a) normal bladder tissue, (b) noninvasive urothelial neoplasm, and (c) invasive urothelial carcinoma. To address the significant class imbalance, especially the underrepresentation of normal tissue, downsampling was implemented for both noninvasive and invasive categories to create a balanced dataset for training and evaluation. Downsampling reduced the overall amount of data but preserved its authenticity while directly balancing class distributions. This approach addresses the problem of class imbalance in a simple and intuitive manner, without the need for complex weighting adjustments, data augmentation, or synthetic data generation, thereby offering advantages in reproducibility and interpretability. Although we fully considered alternative methods, we ultimately chose downsampling to minimize bias, ensure training stability, and enable fair comparisons across classes.

**Fig. 9:**
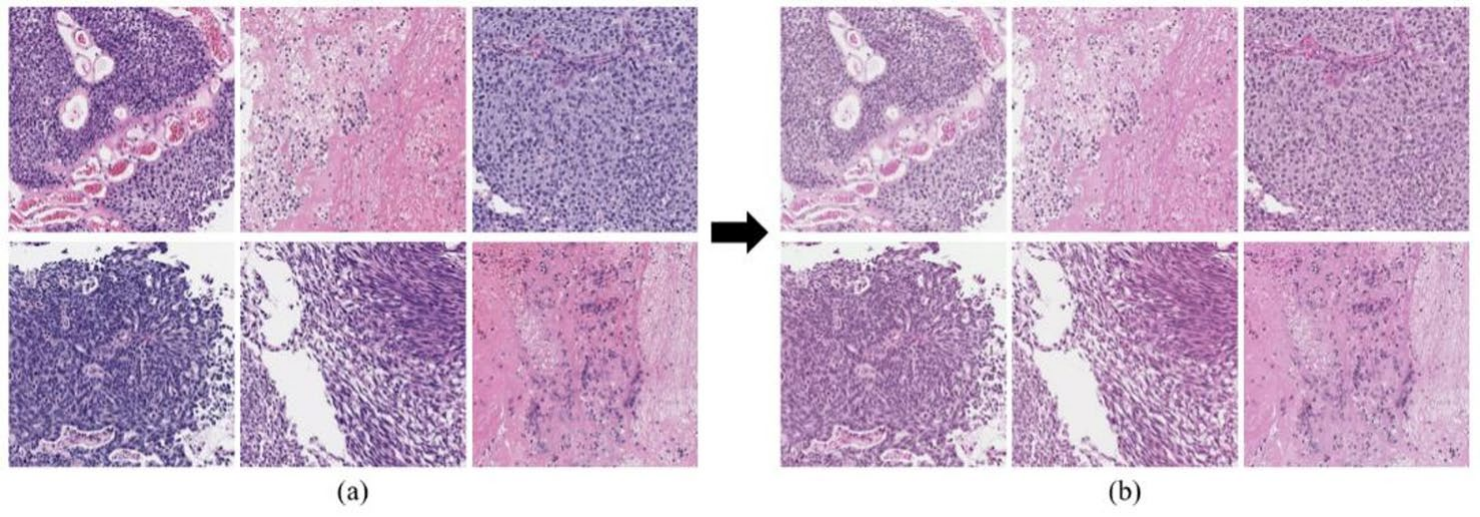
Comparison of image patches before and after stain normalization via the Macenko method: (**a**) original patches prior to stain normalization, and (**b**) corresponding patches after stain normalization.

Owing to the inherent variability in pathology data, H&E-stained slides often exhibit considerable color differences arising from factors such as staining duration, cell density, tissue composition, and section thickness^27^. This variation in color distribution across image patches can negatively affect the performance and stability of deep-learning models. To mitigate this issue, stain normalization was applied using the Macenko method, implemented via the Staintools Python library^28^. A reference patch with high-quality and well-preserved staining was selected as the normalization target, and all patches were adjusted accordingly to achieve a consistent color profile. Figure 9 shows the effect of stain normalization: (a) original patches prior to normalization and (b) corresponding patches after normalization via the Macenko method. As demonstrated, the color tone across all six patches became substantially more uniform, thereby reducing potential sources of variability during training and facilitating a more robust model performance.

The dataset was divided for training via a 5-fold cross-validation approach. During the process of evenly splitting the data into five folds, stratification by institution was applied to ensure that the distribution of each institution’s data was represented consistently across all folds. This prevented data from any single institution from being concentrated in a particular fold and ensured that the characteristics of the multi-institutional dataset were consistently reflected throughout the cross-validation process.

In each experiment, one-fold was designated the test set, whereas the remaining four folds were used for training and validation. This procedure was repeated across all the folds, resulting in a total of five training and evaluation cycles. By doing so, we minimized performance variability due to data imbalance and enabled an objective assessment of the model’s generalization ability. Finally, the performance metrics obtained from each fold were averaged and reported as the final performance of the model.

### Deep learning-based classification model training

In this study, ResNet-50, DenseNet-121, EfficientNet-B6, and Vision Transformer (ViT) were used to classify patch-level data into normal, noninvasive, and invasive categories. In deep neural networks, increasing the model depth often leads to gradient vanishing or explosion problems. To address this issue, we used the ResNet-50 model, which adopts a residual learning framework. The key concept of residual learning in ResNet-50 is that each layer learns a residual function on the basis of the input rather than learning the desired output directly.

The advantage of this approach is that it allows the model to learn the difference between the input and output of each layer, thereby facilitating the optimization of deeper networks. In this framework, the existing information from earlier layers is preserved, and the model learns by adding new information. This enables stable training even as the model depth increases, because learning is performed on the basis of information from the preceding layer^29^. The DenseNet model adopts a feed-forward approach in which all layers are directly connected to one another. In DenseNet, each layer retains all the feature maps from the previous layers and adds newly learned information on top of them. Because of its feed-forward nature, each layer receives the collective features of all preceding layers as inputs and passes its own features to all subsequent layers. This architecture prevents the model from redundantly relearning already captured features, thereby maximizing the training efficiency. As a result, DenseNet is designed with fewer parameters than traditional CNN models, while maintaining or improving performance^30^. Previous studies have rarely addressed all three dimensions of model scaling—depth, width, and resolution—simultaneously. To overcome this limitation, the EfficientNet model was proposed. EfficientNet employs a technique called compound scaling that uniformly scales the depth, width, and resolution using fixed scaling coefficients. In this context, depth refers to increasing the number of layers to make the model deeper, width involves expanding the number of filters in each layer to generate more feature maps, whereas resolution means increasing the input image resolution to capture more detailed pixel information. Compound scaling adjusts these three aspects in a balanced manner by recognizing their interrelated nature. EfficientNet models are available in different versions, depending on the input image resolution. Among them, the patch size used in this study was most closely aligned with the recommended input size of the EfficientNet-B6 model^17^. Whereas the original transformer architecture has been widely used in the field of natural language processing (NLP), it has recently been adopted in the vision domain to leverage its advantages, leading to the development of the Vision Transformer (ViT) model. Transformer-based models offer several benefits, including structural efficiency in capturing and learning global contextual information, rather than mere reduction in computational cost, and independence from the input sequence length^31,32^. The ViT model operates as follows. The input image is divided into fixed-size patches. These patches are then flattened via linear projections and used as patch embeddings. Learnable class and positional embeddings are added to the patch embeddings to facilitate classification. These combined embeddings are fed into a transformer encoder, and the final predicted class is produced through a multilayer perceptron (MLP) head^33^.

The same hyperparameters were applied across all four models for training and validation. The input image size was set as 512 × 512 pixels. Training was conducted for 100 epochs with a batch size of 32, learning rate of 0.0001, the Adam optimizer, and cross-entropy loss function. The models pretrained on ImageNet were loaded via the PyTorch library, and training and validation were performed based primarily on the PyTorch framework. An early stopping mechanism was implemented to prevent overfitting. In addition, the ReduceLROnPlateau scheduler was used to dynamically adjust the learning rate by monitoring the validation loss throughout training.

Visual representation of the deep-learning model predictions was performed using class activation maps (CAMs). The CAM uses the gradients of a specific class to generate a localization map that highlights important regions based on the values computed at the final convolutional layer. In other words, the CAM visualizes the basis of the model’s prediction for a given class on the input image in the form of a heatmap. For the transformer-based model, ViT, the attention values were computed for each patch within the transformer layers. On the basis of these values, CAMs were generated. Unlike CNN-based models, the ViT model displays highly important patches in red and relatively less important patches in blue, thereby offering a distinct visualization of model attention. Cross-validation was conducted to derive generalized performance metrics for the dataset. For cross validation, the entire dataset was divided into *n* folds, and training and validation were performed iteratively in a rotating manner. This validation method allows the extraction of more generalized performance indicators because it does not rely on a single fixed validation set. In the experiments, 5-fold cross-validation was used, in which the dataset was divided into five subsets for validation. UMAP was used for the final visual evaluation. UMAP is a dimensionality reduction method capable of capturing nonlinear structures in high-dimensional data^34^. For the final visual evaluation, UMAP was employed with a model that achieved relatively superior performance. This analysis aims to validate whether the model can effectively distinguish image classes based on meaningful feature distributions. The UMAP was generated using the features extracted from the deepest layer of the high-performance model.

### Statistical analysis

To determine whether there were statistically significant differences among the normal, noninvasive urothelial neoplasm, and invasive urothelial carcinoma groups based on the collected data, p-values were obtained. One-way analysis of variance (ANOVA), a statistical method used to test whether the differences in group means were statistically significant, was applied^35^. To validate the performance of the classification model for normal tissue, noninvasive urothelial neoplasms, and invasive urothelial carcinoma, the predictions of the classification model were compared with the region labels defined by board-certified pathologists. On the basis of this comparison, a confusion matrix was generated and defined as true positive (TP), false negative (FN), false positive (FP), or true negative (TN). The performance metrics used to evaluate the classification results were the Accuracy, Precision, F1 score, and Area Under the Curve (AUC). Accuracy was calculated using the formula (TP + TN)/(TP + TN + FP + FN), which represents the proportion of correctly predicted results. The precision, defined as the proportion of actual positives among all positive predictions, was calculated as TP/(TP + FP). The F1 score, which is the harmonic mean of the precision + recall, provides an overall measure of the model performance and is calculated as 2 × (precision × recall)/(precision + recall). Because this study involved multiclass classification with an imbalanced dataset, a weighted average was used to calculate the metrics. The AUC represents the area under the ROC curve, which illustrates the relationship between the TPR and FPR, and was calculated using the micro-average. As 5-fold cross-validation was used in this study, the performance was evaluated for each fold, and the average was computed. The 95% CI of the mean was calculated to determine the final predictive performance.

### Data Availability

The publicly available Pathology image data of genitourinary cancer Dataset can be accessed through AIHub, a Korean national AI data portal, at the following URL: https://www.aihub.or.kr/aihubdata/data/view.do?currMenu=115&topMenu=100&dataSetSn=71775 (accessed on June 18, 2025). To access the data, users must first create a free account and agree to the terms of use. Access is granted upon request through the AIHub system. This dataset includes annotated whole slide images intended for the development and validation of AI-based models for the analysis of genitourinary cancer pathology.

### Code Availability

The source code is available at: https://github.com/user-dynamite/Digital-pathology-bladder-classification.
